# RNAi Therapeutic Platforms for Lung Diseases

**DOI:** 10.3390/ph6020223

**Published:** 2013-02-06

**Authors:** Yu Fujita, Fumitaka Takeshita, Kazuyoshi Kuwano, Takahiro Ochiya

**Affiliations:** 1 Division of Molecular and Cellular Medicine, National Cancer Center Research Institute, Tokyo, 104-0045, Japan; E-Mails: yufujit2@ncc.go.jp (Y.F.); futakesh@ncc.go.jp (F.T.); 2 Division of Respiratory Diseases, Department of Internal Medicine, Jikei University School of Medicine, Tokyo, 105-8461, Japan; E-Mail: kkuwano@jikei.ac.jp (K.K.)

**Keywords:** RNAi, siRNA, miRNA, drug delivery system, lung diseases, lung cancer

## Abstract

RNA interference (RNAi) is rapidly becoming an important method for analyzing gene functions in many eukaryotes and holds promise for the development of therapeutic gene silencing. The induction of RNAi relies on small silencing RNAs, which affect specific messenger RNA (mRNA) degradation. Two types of small RNA molecules, *i.e.* small interfering RNAs (siRNAs) and microRNAs (miRNAs), are central to RNAi. Drug discovery studies and novel treatments of siRNAs are currently targeting a wide range of diseases, including various viral infections and cancers. Lung diseases in general are attractive targets for siRNA therapeutics because of their lethality and prevalence. In addition, the lung is anatomically accessible to therapeutic agents via the intrapulmonary route. Recently, increasing evidence indicates that miRNAs play an important role in lung abnormalities, such as inflammation and oncogenesis. Therefore, miRNAs are being targeted for therapeutic purposes. In this review, we present strategies for RNAi delivery and discuss the current state-of-the-art RNAi-based therapeutics for various lung diseases.

## 1. Introduction

RNA interference (RNAi) is a natural endogenous mechanism for silencing gene expression that, recently, has been the focus of considerable attention for its potential use in new drugs [1]. The expression of a specific gene can be regulated using different mediators, such as short hairpin RNA (shRNA), microRNA (miRNA), and small interfering RNA (siRNA). Gene silencing can be induced by siRNAs through a sequence-specific cleavage of perfectly complementary messenger RNA (mRNA); in contrast, miRNAs mediate translational repression and transcript degradation for imperfectly complementary targets. RNAi-based therapy may provide several advantages over conventional therapeutic approaches using small molecules, proteins, and monoclonal antibodies. Unlike traditional drugs, RNAi-based therapeutics can inhibit all classes of gene targets with high selectivity and potency, can provide personalized therapy, can be easily synthesized, and can be conducted through rapid steps of lead identification and optimization [2]. Synthetic oligonucleotides have other potential advantages, such as drug-like properties, that can often be improved through the introduction of chemical modifications, and manufacturing processes are usually amenable to scaled-up production. Several *in vivo* studies in animal models have demonstrated that RNAi-based therapeutics are effective for the treatment of various diseases, such as viral hepatitis [3], Huntington's disease [4], and some cancers [5]. Furthermore, there are several RNAi therapeutic agents in clinical development. Nevertheless, previous investigations have shown that there are several obstacles that need to be overcome before routine clinical applications are made. RNAi-based therapeutics are promptly degraded by nucleases when they are administered systemically, and chemical modifications at specific positions or formulation with delivery vectors have been shown to improve stability, but they may attenuate the suppressive activity of oligonucleotides [6]. Their systemic administration may induce undesirable off-target effects by activating the innate immune system via toll-like receptor (TLR)-dependent or independent mechanisms, leading to an increased number of inflammatory cytokines [7]. Success of the delivery of RNAi-based therapeutics necessitates efficiency, convenience, and patient compliance of the delivery route. For this reason, direct administration of RNAi-based therapeutics into the target organs is a promising approach for overcoming the problems of systemic administration. So far, an approach for drug treatment has been developed that includes transdermal, rectal, vaginal, and pulmonary drug delivery systems.

The lung is susceptible to many diseases because of its location and physiological function. It is usually exposed to many environmental pollutants, including smoke and volatile organic compounds, which lead to diseases such as asthma, emphysema, and lung cancer. Furthermore, many of the lethal infectious diseases are airborne and use the lungs as their main entrance to the body. Therefore, lung diseases have received particular attention as targets of direct administration of RNAi-based therapeutics. As a direct route to the lung, pulmonary delivery has offered a new method for the treatment of various lung diseases, such as cancer [8,9,10,11,12], respiratory infectious diseases [13,14,15,16,17], asthma [18,19], and pulmonary fibrosis [20,21]. The approach could potentially enhance the retention of RNAi-based therapeutics in the lungs and reduce systemic toxic effects. However, the development of pulmonary delivery for clinical applications remains a challenge for research of drug delivery systems and development. This review focuses on the latest development of pulmonary delivery and future plans for the RNAi-based treatment of various lung diseases.

## 2. Delivery of RNAi-Based Therapeutics to the Lungs

The lung is emerging as an attractive target for the treatment of various pathogenic disorders using RNAi-based therapeutics because of the increasing incidence of lung diseases with high mortality and morbidity. The primary obstacle to translating RNAi-based therapy from the laboratories into the clinics is delivery. Delivery of siRNAs to the lungs is often studied and described using different routes and delivery strategies [22]; therefore, the focus of this chapter is on the characteristics of siRNA delivery to the lung.

In general, lung targeting can be achieved by intravenous as well as intrapulmonary administration. Although multiple routes of administration using siRNAs have been used, ranging from direct injection into target tissues to systemic administration, the use of siRNAs for the treatment of respiratory diseases has tended to focus on direct intratracheal or intranasal delivery of siRNAs to the lungs. The direct route offers several important benefits over systemic delivery, including the requirement for lower doses of siRNAs, the reduction of undesirable systemic side effects, and improved siRNA stability due to lower nuclease activity in the airways than in the serum. Lastly, and most importantly, in the context of treating respiratory disease, local administration of siRNAs allows direct access to lung epithelial cells, which are important cell types in a variety of pulmonary disorders [23]. Since the lung is accessible to therapeutic agents via multiple intrapulmonary routes, it has been a convenient model for *in vivo* validation of siRNA-mediated therapeutic gene silencing.

### 2.1. Pulmonary Delivery Approaches

Pulmonary delivery of therapeutic molecules, such as proteins and peptides, has been investigated for more than 30 years [23]. Pulmonary delivery can be achieved using intratracheal, intranasal, and inhalation routes. In most of the pulmonary siRNA therapy studies *in vivo*, siRNAs were delivered intratracheally or intranasally. In particular, intranasal delivery of siRNAs is widely used for administration due to its simplicity and adaptability to the delivery of various siRNA formulations, such as nasal spray and droplets. Although administration by inhalation is clinically the most common and non-invasive method to deliver therapeutic agents into the lung, only a few animal studies have been conducted on the formulation of inhalation of siRNAs [24,25].

#### 2.1.1. Intratracheal and Intranasal Delivery

**Figure 1:**
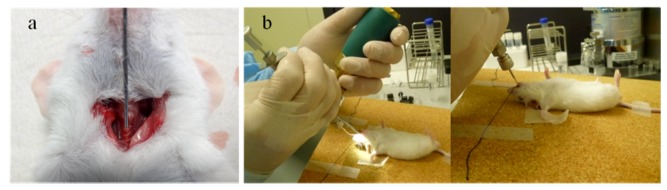
Intratracheal route of siRNA administration into the lungs *in vivo* studies. (**a**) Intratracheal route: under anesthesia, the trachea is exposed surgically, and a tube or needle is inserted through an incision made between the tracheal rings. Complications, such as vascular injury and air leakage, are possible due to the tracheotomy. (**b**) Endotracheal route: siRNAs are sprayed directly from the mouth into the lungs using a MicroSprayer^® ^aerolizer (Penn-Century, Philadelphia, PA, USA) and a laryngoscope. It is important to maintain a clear view of the trachea during the procedure.

Intratracheal administration is one common method of pulmonary drug application. The pulmonary application method can be useful for the study of drug and vaccine delivery to the airway and lungs. Many animal studies have relied on intratracheal delivery of siRNAs to the lungs [15,26,27,28,29,30,31]. Moreover, some of them have also reported successful delivery of unmodified siRNAs without delivery vectors. The advantages of the intratracheal route are that it ensures high delivery efficiency with minimal loss of the drug and the application itself is quick and relatively inexpensive. The disadvantage is that, because it requires a surgical procedure, such as a tracheotomy, it is not a comfortable delivery method from the patient's viewpoint. With this method, the trachea is exposed during the procedure, and an endotracheal tube or microsyringe is inserted through an incision between the tracheal rings [Figure 1(a)]. This method is not routinely used for drug administration in humans [32]. On the other hand, Bivas-Benita *et al.* reported a relatively non-invasive pulmonary delivery via the endotracheal route [33]. In this method, the formulation is sprayed under anesthesia from the mouth to the trachea of mice using a microsyringe (Figure 1b). The main benefit of the endotracheal application is the visualization of the trachea, which is important for reliable lung administration. Compared with traditional surgery, Bivas-Benita *et al.* reported that no mortality occurred as a result of the use of the endotracheal technique. Endotracheal applications are currently being used by many practitioners in the pulmonary field [22,34]; this is useful for studying pulmonary drug delivery in mice. However, the approach is more complex in humans because an artificial path for the delivery of drugs into the lungs is used. Therefore, the method is being used in animal models to test and evaluate its reliability for possible clinical applications.

Intranasal delivery is another common method of pulmonary drug application in animal studies. In many studies, *in vivo* success has been demonstrated in delivering siRNAs to the lungs intranasally [22,35,36]. An experimental setup of intranasal delivery by spray or droplet is simple and painless for the animal. Although the success in delivering siRNAs intranasally in rodents cannot be completely extrapolated to human use because of the significant differences in lung anatomy [37], this approach has potential for the clinical application of siRNAs. Phase II clinical trials have been initiated for the treatment of respiratory syncytial virus (RSV) infection, making use of intranasal application of naked chemically modified siRNA molecules that target viral gene products [17,38] (see Section 3.1.1. for details).

Intranasal entry has long been used to administer small molecules, such as proteins, for systemic delivery. Because the nasal mucosa is highly vascularized, delivery of a thin epithelium of medication across the surface area can result in rapid absorption of the medication into the blood. Therefore, siRNAs administered intranasally might be deposited in the nose, and some of them may be unable to reach the lower respiratory tract. In fact, it has been reported that intranasal application of unformulated siRNAs resulted in lower delivery efficiency and homogeneous pulmonary distribution than that achieved with intratracheal application [31]. The intranasal method is suitable for some lung diseases, such as upper respiratory infection by RSV, and it also has potential for systemic delivery rather than pulmonary delivery of siRNAs. Therefore, it is important to consider the route of administration in animal studies when assessing the delivery and therapeutic efficacy of a formulation for pulmonary delivery. Careful choice of efficient delivery in response to the condition of lung diseases is necessary.

#### 2.1.2. Inhalation Delivery

The use of aerosols to deliver medication to the lungs has a long history. Administration by inhalation is a popular and non-invasive method of delivering agents into the lungs. There are several inhalation devices available for the delivery of drugs into the lungs. Metered dose inhalers (MDIs) and dry powder inhalers (DPIs) are the most common modes of inhaled delivery. MDIs are the most commonly used inhalers for several lung diseases, such as asthma, bronchitis, and chronic obstructive pulmonary disease (COPD), and a spacer is an external device that is attached to an MDI to allow for better drug delivery by enhanced actuation and inhalation coordination. For most MDIs, the propellant is one or more gases called chlorofluorocarbons (CFCs). Although CFCs in drugs are safe for patients to inhale, they are harmful to the environment. Therefore, further development of inhalable siRNAs may not be the best way forward. DPIs are devices that deliver medication to the lungs in the form of dry powder. The use of DPIs has already shown promise for the *in vivo* delivery of therapeutic macromolecules such as insulin [39] and low-molecular-weight heparin [40]; thus, it could be a better device for delivering siRNAs to the lungs. The advantages of DPIs are improved stability and sterility of biomolecules over liquid aerosols and propellant-free formation.

Although drugs are commonly delivered to the lungs by inhalation, most *in vivo* studies using siRNAs have relied on intratracheal or intranasal delivery. The reason could be the difficulty in formulating inhalable siRNAs and maintaining the stability during the delivery process. A suitable carrier is also needed to protect nucleic acids from degradation due to shear force and increased temperature during the drying process. The use of spray-drying as a technique for engineering dry powder formulations of siRNA nanoparticles, which might enable the local delivery of biologically active siRNA directly to the lung tissue, has been demonstrated [24,25]. In the future, the technique is desirable to estimate the *in vivo* study on siRNA therapy for inhalation. In the long term, we anticipate that there will be more sophisticated devices for clinical use and that those currently being developed will be more suitable.

### 2.2. Extracellular and Intracellular Barriers to siRNA Delivery

**Figure 2:**
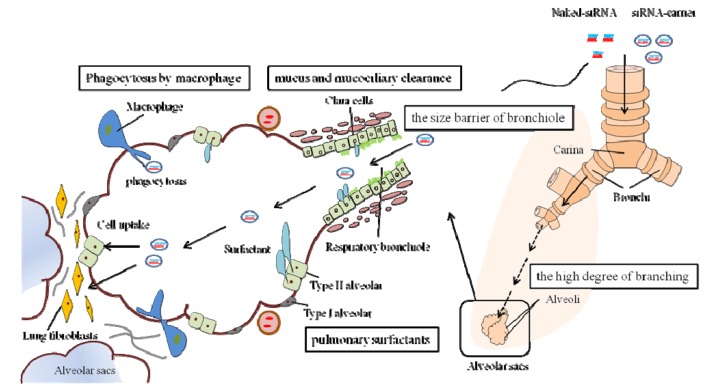
Extracellular barriers to pulmonary siRNA delivery. The anatomical feature of the respiratory tract is its high degree of branching. The mucus lines the respiratory epithelium from the nasal cavity to the terminal bronchioles. The deposited particles on the ciliated epithelial cells are rapidly cleared by the mucociliary clearance actions. Mucus and mucociliary clearance of mucus-trapped particles is a pulmonary defense mechanism as a physiological barrier. In the alveolar, clara cells and type II alveolar cells secrete on the surface of the alveolar epithelium, forming a thin layer of pulmonary surfactants. The surfactants act as the main barrier for siRNA delivery because they reduce the transfection efficiency. In addition, the macrophages located in the alveoli rapidly engulf the foreign particles by phagocytosis. The particles taken up into the macrophages are subsequently degraded inside the cells. These factors present major barriers to targeted pulmonary delivery.

There are two main barriers to efficient pulmonary siRNA delivery to the cells of the lung. The first is the complex, branched anatomy of the lungs and biomechanical barriers, such as the mucus layer covering the airway cells [41,42] (Figure 2). A remarkable feature of the respiratory tract is its high degree of branching. Airway consists of respiratory bronchioles, alveolar ducts, and alveolar sacs. All of these structures bear alveoli, the tiny air sacs in which the gas exchange takes place. It is generally acknowledged that the critical factor for efficient siRNA delivery depends on the properties of RNAi drug particles in terms of size, charge, shape, velocity and density. For efficient pulmonary siRNA delivery, the particles must be deposited in the lower respiratory tract. Deposition in the airway is affected by the particle size and patient's pulmonary function. A particle size between 1–5 μm is found to be the most appropriate for deposition at the lower respiratory tract [23]. In addition, the presence of mucus and surfactant proteins, the mucociliary clearance actions, and phagocytosis by macrophages present major barriers to targeted pulmonary delivery. Therefore, delivery systems usually require delivery vectors, and these vectors need to be designed in order to maximize the siRNA deposition to the diseased area of the respiratory tract. Besides, the extracellular barriers to siRNA delivery also depend on physiological features of the respiratory tract, which may change with the disease stage and characteristics of the patient. At the active stage of lung disease, the physiological conditions of the airways might change and have significant impact on the efficiency of the pulmonary delivery system. During infection, inflammation, and allergic reaction, there is an increase in mucus secretion along with the impaired mucociliary clearance [43]. Moreover, asthma and COPD are both chronic inflammatory conditions of the lung associated with structural “remodeling” that is inappropriate to the maintenance of normal lung function [44]. The airway wall thickness, the high viscosity, and the composition of the mucus layer might be altered in patients who have inflammatory lung diseases.

**Figure 3:**
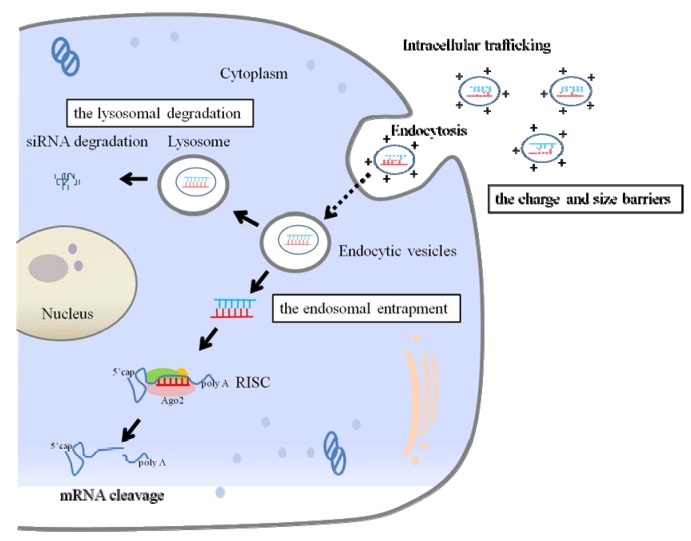
Intracellular barriers to pulmonary siRNA delivery. Barriers to cellular internalization are dependent on the surface properties of siRNA and carriers (e.g., charge and size). After siRNAs are successfully taken into the target cells by endocytosis, the main barriers for delivering siRNAs to its site of action are the endosomal entrapment and lysosomal degradation of siRNA and carriers. To direct target-gene silencing, the siRNAs need to escape from the endosome into the cytoplasm, where they associate with the Ago2/RNA-induced silencing complex (RISC) to direct the cleavage of mRNAs bearing complementary binding sites.

The second is the airway cell membrance and its intracellular barriers (Figure 3). For efficient gene silencing in the lungs, siRNAs must be delivered to their site of action, be stable, enter the target cells, and be present in the cytoplasm at sufficient concentration. Once the siRNAs reach the target cells, they must be trafficked into the cytoplasm and taken up by Argonaute (Ago)2/RNA-induced silencing complex (RISC), which degrades mRNAs and, subsequently, suppresses the sequence-specific gene expression. For efficient endocytosis to occur, particles should be under 150 nm in size. Particles within this size range could also avoid macrophage uptake and delayed lung clearance [45]. The physicochemical properties of siRNAs also play a significant role in crossing the biological membrane. Despite their small size, the negative charge and chemical degradability of siRNA molecules prevent them from readily crossing biological membranes. Therefore, efficient siRNA delivery approaches need to overcome this limitation by facilitating cellular uptake. One of the main functions of a delivery vector is to facilitate the cellular uptake of siRNAs [46]. The electrostatic complexation of siRNA molecules with cationic lipids and polymers helps to mask their net negative charge. The positively charged siRNA carrier complex interacts with anionic proteoglycans on the cell membrance, forms an endocytic vesicle, and enters the cells by endocytosis [47]. After cellular internalization, the siRNA carrier complex in endocytic vesicles is transported along microtubules to lysosomes that are co-localized with the microtubule-organizing center. To avoid lysosomal degradation, siRNAs must escape from the endosome into the cytoplasm, where they can associate with the RNAi machinery. Endosomal escape is a major barrier for efficient siRNA delivery [48,49]. The endosomal entrapment and lysosomal degradation of siRNA and carriers contribute to the low transfection efficiency and is a major difficulty for delivery vectors. An ideal delivery agent should protect siRNAs from enzymatic degradation, facilitate cellular uptake, and promote endosomal escape inside the cells with negligible toxicity.

### 2.3. Delivery Method of siRNA to the Lungs

Multiple approaches for the delivery of siRNAs have been reported, ranging from the relatively simple direct administration of saline-formulated siRNAs to lipid-based and polymer-based nanoparticle approaches and siRNA conjugation and complexation approaches [50]. The negative charge and chemical degradability of siRNAs under physiologically relevant conditions make its delivery a major challenge. Accordingly, the delivery of siRNAs usually requires a vector or carriers for their transfection into the target cells. In general, both viral and non-viral vectors are being assessed for siRNA delivery to lung cells. Some viral vectors, such as retroviruses and adenoviruses, have been demonstrated to mediate gene silencing in an *in vitro* lung model [51] and to induce RNAi in a range of animal tissues [52]. Recently, Guo *et al.* showed that lentivirus-mediated siRNA was used to specifically knock down the expression of nuclear protein 1 (NUPR1) *in vivo*, which resulted in inhibited tumor growth [53]. However, viral-based delivery has several disadvantages. The immune response to viruses not only impedes gene delivery but also has the potential to cause severe complications [54]. Recent well-documented cases, such as the death of Jesse Gelsinger due to complications related with an adenoviral delivery vector, highlight this problem [55]. In addition, some viral vectors may insert their genome at random positions in the host chromosome, which eventually restrict the gene function [56].

As an alternative to viral vectors, non-viral vectors, including lipid and polymer-based vectors, have been generally used for the delivery of siRNAs to the lungs due to their reduced toxicity [57]. Ongoing research into the transfection of primary cells and whole organisms with siRNA using non-viral transfection agents has produced some promising results. Lipid-based delivery vectors are successfully used to deliver siRNA *in vitro* and *in vivo* [58]. Cationic lipids are composed of positively charged head, a linker and hydrophobic. In general, lipid-based complexes are easy to formulate and good transfection efficacy is achieved due to interaction with negative charged cell membrance. Many commercial siRNA transfection agents are lipid-based delivery system, some of which are also employed for pulmonary delivery—DharmFECT [30], Oligofectamine [59], Lipofectamine [60] and TransIT-TKO [35]. Similarly, cationic polymers have also been assessed for siRNA delivery to lung cells. Cationic polymer polyethylenimine (PEI) is widely used for siRNA delivery [13,61]. PEI is considered as the gold standard for *in vitro* gene delivery and its transfection efficiency depends on the molecular weight and degree of branching.

On the other hand, lipid-based vectors can also induce toxicity and non-specific activation of inflammatory cytokine and interferon responses [62,63]. Although polymer-based vectors elicit a relatively less strong immune response than lipid-based vectors, effective siRNA delivery to a local area in lung diseases requires more attention to the development of non-toxic delivery vectors. An important point for siRNA-mediated inhibition of gene expression is whether the observed effects are specific rather than due to off-target effects and free from potential interferon responses [64,65]. Interestingly, some studies have shown that it was possible to administer “naked siRNAs” to mice and down-regulate an endogenous or exogenous target without inducing an interferon response [66].

The term “naked siRNAs” refers to the delivery of siRNAs without any delivery vectors. Naked siRNAs are degraded by serum endonucleases and are usually removed by glomerular filtration, resulting in a short plasma half-life of < 10 min. Thus, some studies of systemic delivery of naked siRNAs have failed to achieve the downregulation of the targeted gene [67,68]. In contrast, there have also been some successes of locally delivering naked siRNAs to the lungs [15,16,20,31]. A few of them reported that the use of delivery vectors showed no significant difference in gene silencing efficiency compared to that of naked siRNAs [16,35]. Indeed, in one clinical trial, the delivery of naked siRNAs for the treatment of RSV has been used [17,38]. This successful evidence can be because that naked siRNAs for clinical applications are highly chemically modified to prevent nuclease-induced degradation and presumably minimize immune stimulatory effects. Although it is unclear how the naked siRNAs cross the cell membrane, gain access to the cytoplasm, and remain intact to perform their biological action, both animal and human trials have been conducted successfully, showing the efficacy of naked siRNAs (ALN-RSV01) that were administered intranasally. This explanation has not been confirmed, but the physiological damage of respiratory epithelial cells caused by viral infection may have possibly influenced the mystery. The active change in airway epithelial cell membrance caused by infectious disease might affect cellular internalization. Naked siRNA delivery has some advantages, such as simple formation and the absence of toxicity or inflammatory responses that are usually associated with delivery vectors. Nevertheless, the advantage of naked siRNAs over delivery vectors in the treatment of lung diseases is controversial [69,70]. Further *in vivo* investigations about both naked siRNAs and non-viral vectors are required.

## 3. RNAi Medicine in Lung Diseases

Lung disease is a major cause of death, and diminished quality of life is responsible for the suffering of many patients. Various lung diseases make life extremely difficult for the patients, and severe cases of these lung diseases can result in death. The high death rates associated with lung cancer are partially due to the fact that it is unfortunately difficult to cure. Above all, COPD is the fourth-leading cause of death in most industrialized countries and is predicted to become third by 2020 [71]. Therefore, decisive action is needed to stem the rising health and economic burden this represents. Chronic lung diseases, such as COPD and asthma, are disorders of the airways largely related to the presence of persistent inflammation. The approval of inhaled corticosteroids pioneered a new generation of therapy in treating chronic inflammatory diseases. This was the first time that an anti-inflammatory product was available to reduce the characteristic lung inflammation in airways and the associated obstruction. Corticosteroids are still an important therapeutic intervention. However, they are used with limitations in COPD and moderate to severe asthma. Likewise, the treatment of various refractory lung diseases also depends on systemic corticosteroid therapy. Many of these patients also suffered various side effects from systemic corticosteroid use, such as weight gain and uncontrolled hyperglycemia. Treatment of lung disease using cell-specific targeting as well as RNAi techniques represents a novel strategy and could possibly provide new opportunities in nanomedicine. Pulmonary applications of siRNA in *in vivo* conditions are frequently studied and often result in clinical trials [57,72]. The findings of recent clinical studies of pulmonary RNAi therapeutics are discussed.

### 3.1. Therapeutic siRNAs for Lung Diseases

Since the discovery of RNAi, the therapeutic potential of siRNAs has been rapidly recognized. In 2004, the first human clinical trial of RNAi-based therapy was initiated for the treatment of age-related macular degeneration with a siRNA targeting VEGF-receptor 1 delivered intravitreally [73]. Many studies have been conducted over the past few years that involve the delivery of siRNAs to the lungs for the treatment of various lung diseases. Delivery to the lungs will be most important to moving siRNA technology into the clinic. A number of siRNA-based therapies are being evaluated in clinical trials for the treatment of different conditions, including lung diseases such as asthma and RSV infection. Table 1 is a summary of clinical trials of siRNA-based therapeutics [74].

**Table 1:** Summary of siRNA-based therapeutics in clinical trials.

Drug	Route of Administration	Delivery Agent	Disease	Target	Stage of Clinical Trial
Excellair^TM^	Inhalation	Unknown	Asthma	Syk kinase	II
ALN-RSV01	Intranasal spray	Naked siRNA	RSV infection	RSV nucleocapsid	IIb
Atu027	IV	Lipid nanoparticles	Advanced solid cancer (Metastatic lung cancer)	PKN3	I
TKM-ApoB	IV	Lipid nanoparticles	Hypercholesterolemia	ApoB	I
TKM-PLK1	IV	Lipid nanoparticles	Cancer	Polo-like-kinase1	I
ALN-VSP02	IV	Lipid nanoparticles	Solid cancers with liver involvement	KSP and VEGF	I
ALN-TTR01	IV	Lipid nanoparticles	Transthyretin-mediated amyloidosis (ATTR)	Transthyretin (TTR)	I
CALAA-01	IV	Cyclodextrin nanoparticles	Solid tumor	RRM2	I
siG12D LODER	EUS biopsy needle	Miniature biodegradable polymer matrix	Pancreatic ductal adenocarcinoma	KRAS	I
I5NP	IV	Naked siRNA	Acute kidney injury	p53	I/II
QPI-1007	IVT	Naked siRNA	Glaucoma and acute eye injury	Caspase-2	I
TD101	Intradermal injection	Naked siRNA	Pachyonychia congenita	Keratin 6a N171Kmutant mRNA	Ib
SYL040012	Ophthalmic drops	Naked siRNA	Ocular hypertension and glaucoma	Adrenergic receptor beta-2	I/II
AGN-745	IVT	Naked siRNA	AMD	VEGF-receptor1	II
PF-655	IVT	Naked siRNA	AMD and diabetic macular edema	RTP801 (pro-angiogenic factor)	II
Bevasiranib	IVT	Naked siRNA	AMD	VEGF	III

IV: Intravenous injection; IVT: Intravitreal injection; RSV: Respiratory syncytical virus; AMD: Age-related macular degeneration; Syk: spleen tyrosine kinase; PKN3: protein kinase N3; KSP: kinesin spindle protein; RRM2: M2 submit of ribonucleotidereductase; KRAS: V-ki-ras2 Kirsten rat sarcoma viral oncogene homolog; VEGF: vascular endothelial growth factor.

#### 3.1.1. Pulmonary Viral Infections

SiRNA shows potential for the treatment of various pulmonary viral infections, and it has been reported that siRNA-based therapeutics can also be used in the treatment of influenza [13], parainfluenza virus [35], severe acute respiratory syndrome (SARS) [14], and RSV [35]. Above all, RSV is the most promising therapeutic target of siRNAs.

RSV is a common cause of serious respiratory infections in infants and children. It also produces significant morbidity and mortality in adult immunocompromised or elderly populations [75]. An RSV vaccine is not available, and the only approved antiviral therapy for RSV is undesirable for pediatric patients due to its potential teratogenicity and limited effectiveness. Thus, a safe and efficacious RSV therapy has long been awaited for both pediatric and adult patients. RNAi-based therapy has shown promising effects in murine models of RSV infection [35]. The siRNA, ALN-RSV01, is directed against the mRNA encoding the N-protein of RSV that exhibits specific *in vitro* and *in vivo* anti-RSV activity. It is delivered without a delivery vector as a nasal spray and targets the upper respiratory tract instead of the lower lung area. ALN-RSV01 has undergone complete phase I intranasal and inhalation studies in healthy adults and has been found to be generally well tolerated [38]. Additionally, ALN-RSV01 has been evaluated in a randomized, double-blind, placebo-controlled phase II trial in lung transplant patients with RSV respiratory tract infection [76]. The administration of ALN-RSV01 to RSV infected lung transplant patients was safe and well tolerated and associated with a statistically significant improvement in symptoms. Based on these results, a larger multinational, randomized, double-blind Phase IIb trial of ALN-RSV01 has been initiated in lung transplant patients to confirm and extend these findings.

#### 3.1.2. Lung Cancer

Cancer is a major target of RNAi-based therapy, as oncogenes, mutated tumor suppressor genes, and several other genes contributing to tumor progression are potentially important targets for gene silencing by RNAi. Lung cancer is one of the most frequent tumors worldwide with regard to incidence rates and mortality. Patients with lung cancer are commonly diagnosed at an advanced stage of the disease and have limited therapeutic options. Although the knowledge regarding the genetic and molecular basis of lung cancer has regularly increased, the median survival rates of individuals with advanced lung cancer are still poor.

RNAi-based therapy is an attractive strategy for the development of more effective anticancer therapies with reduced treatment-related toxicity. The major advantage of RNAi therapeutics in cancer might be the simultaneous targeting of multiple genes belonging to different cellular pathways that are involved in tumor progression. The simultaneously inhibition of several genes would also minimize the risk of drug resistance normally encountered with small molecule-based therapies, involving siRNAs and miRNAs. There have already been significant improvements in siRNAs for primary or metastatic lung cancer treatment by targeting oncogenes such as Akt1 [9], Wilms tumor 1 (WT1) [12], overexpressed genes such as the insulin-like growth factor receptor 1 (IGF-1R) [77] , NUPR1 [53] and EZH2 [78]. Some of these studies have successfully shown the efficacy of RNAi-based therapy through intrapulmonary administration of siRNAs with non-viral vectors. Although strategies to minimize off-target and nonspecific immune stimulatory effects must be devised, these data suggest that the silencing of the target gene with siRNAs is an attractive strategy for the prevention and treatment of primary and metastatic lung cancer. There are currently some clinical trials in progress estimating the safety and efficacy of siRNA-based drugs for cancer treatment. Atu027, a siRNA-lipoplex targeted against protein kinase N3 (PKN3), prevented lung metastasis in a phase I trial of various cancer models [79]. PKN3 is a downstream effector of the phosphoinositide 3-kinase (PI3K) signaling pathway [80], which regulates diverse cellular responses, including development, growth, and survival [81]. Recently, PKN3 has also been considered as a suitable therapeutic target for modulating tumor angiogenesis because loss of function analysis with Atu027 in cultured primary endothelial cells showed an essential role of PKN3 for endothelial tube formation and migration [79]. Atu027 can be considered as a potential siRNA for preventing lung metastasis and might be suitable for preventing hematogenous metastasis combined with conventional cancer therapy.

#### 3.1.3. Inflammatory Lung Diseases

Inflammatory lung disease, also called COPD, includes a wide range of lung ailments. These related diseases include asthma, pulmonary fibrosis, and chronic bronchitis. They are influenced by a combination of environmental, genetic, and epigenetic components [82]. COPD is a chronic inflammatory disease of the airways. This disease is hallmarked by airflow that is not fully reversible. Systemic and local airway inflammation has been implicated in the pathogenesis of COPD [83]. COPD is mainly associated with tobacco smoking, and recent studies investigating the pathophysiology of emphysema have demonstrated that cigarette smoke can cause cells to enter cellular senescence. Smoking might cause cells to senesce due to DNA damage through increased cell turnover, which in turn leads to accelerated telomere shortening [84]. Lately, a lot of studies have investigated the role of cellular senescence in the development and progression of COPD [85]. Although several medication classes, including inhaled corticosteroids, are used for COPD treatment, none of these medications have been shown to significantly improve long-term lung function during the progression of the disease. Current interventions that have been shown to improve mortality in COPD are cessation of smoking and delivery of supplemental oxygen when hypoxemia is present.

Many people are developing COPD, and the cause of this condition is complicated and not thoroughly understood. One key factor is genetic susceptibility. Some studies have shown a large genetic contribution to the variability in pulmonary function and COPD [86,87]. Polymorphisms in multiple genes have been reported to be associated with COPD [87], such as transcription factor [e.g. nuclear factor-kappa B (NFκB)] [88], extracellular matrix (e.g., matrix metalloproteinase-12 (MMP-12)) [89,90], cytokines [e.g. tumor necrosis factor (TNF)-α] [91], chemokines [e.g. interleukins (IL)-8, IL-8 receptor and chemokine receptor (CCR)1] [92,93], and apoptosis (e.g., caspase-3 and vascular endothelial growth factor (VEGF)) [94,95]. Many of these have been identified as possible targets for therapeutic intervention using molecule inhibitors or antagonists. Although several new treatments that target the inflammatory process are now in clinical development, such as TNF-α inhibitors and I-kappaB kinase complex 2 (IKK2) inhibitors [96,97], clinical trials with siRNAs have never been performed in COPD. The delay of drug development for COPD might be due to the relatively recent emergence of research addressing the molecular basis of COPD. Furthermore, more research is needed to understand the essential molecular mechanisms about the pathogenesis of COPD and to develop monitoring techniques to support the development of RNAi therapies. Currently, no available treatments reduce the progression of COPD or suppress the inflammation in small airways and lung parenchyma. The RNAi-based approach for the key molecules also has potential implications for the treatment of COPD.

Asthma is also a chronic inflammatory disease of the airways characterized by variable and recurring symptoms and reversible airflow obstruction. The World Health Organization estimates that 300 million people are currently affected and that, by the year 2025, another 100 million will be affected by the disease [98]. Inhaled corticosteroids are very effective in mild asthma because they improve symptoms and decrease exacerbations. However, in moderate and severe asthma, inhaled corticosteroids have important therapeutic limitations. Although corticosteroids remain an important therapeutic intervention for inflammatory lung diseases, their use is not always completely effective and is associated with side effects. Due to such limitations, it is clear that there is a need for new types of medications that can treat and improve the prognosis of moderate to severe asthma.

Many target genes have been identified that participate in the pathogenesis of asthma. The most promising targets include genes coding for cytokines (IL-4, IL5, and IL-13), cytokine and chemokine receptors (IL-4 receptor and CCR3), and tyrosine kinases [spleen tyrosine kinase (Syk) and LCK/YES-related novel tyrosine kinase (Lyn)], as well as for transcription factors [signal transducers and activators of transcription 1 (STAT1), STAT6, GATA3, and NFκB] that are involved in asthma [19,99,100]. The genes that have been assessed as siRNA targets for the treatment of asthma in preclinical models are reported [101]. Currently, in a clinical trial for asthma, Excellair^TM^ (ZaBeCor, Bala Cynwyd, PA, USA), a siRNA that targets Syk, is being used. The kinase is involved in signaling from a B cell receptor and is a key regulator of downstream signaling cascades that ultimately lead to the activation of several pro-inflammatory transcription factors. It has been reported that antisense oligonucleotides administered by aerosol were potent to decrease Syk expression, mediator release from alveolar macrophages, and Syk-dependent pulmonary inflammation [102]. Moreover, inhibition of inflammatory mediators was shown in a study using siRNA targeting Syk in airway epithelial cells [103]. Following the successful results of the company's Phase I clinical trial, a Phase II trial for its asthma drug candidate Excellair^TM^ has already been initiated. Some of the current treatments for asthma and other inflammatory conditions, such as TNF-α inhibitors or leukotriene inhibitors, inhibit only one of the mediators of inflammation. In contrast, siRNA targeting Syk seeks to inhibit an initial signaling step of inflammation and, thereby, prevent the release of multiple inflammatory mediators. Overall, recent progress of siRNAs to the lungs has also improved the therapeutic feasibility of RNAi for inflammatory lung diseases. The rapid progress will put siRNA-based therapeutics on a fast track to the clinic.

### 3.2. Therapeutic microRNA/Anti-microRNA for Lung Diseases

MiRNAs are small endogenous noncoding RNAs that regulate gene expression by repressing translation or promoting the degradation of their target mRNA. MiRNAs regulate gene expression by binding to the 3′ untranslated region (UTR) of their target mRNAs and mediating mRNA degradation or translational inhibition. In the human genome, transcripts of approximately 60% of all mRNAs are estimated to be targeted by miRNAs [104]. According to their function, miRNAs play an important role in cellular processes as development, proliferation, and apoptosis of pulmonary pathologies [105]. A growing number of miRNAs have been shown to be involved in different lung diseases. This evidence makes miRNAs a promising technology for current and future therapeutic development. We discuss the role of some miRNAs in various lung diseases as well as the possible future of these discoveries in clinical applications. Table 2 shows the summary of miRNAs in therapeutic development. At this point, a miRNA-based therapy has already entered a phase II clinical trial.

**Table 2:** miRNAs in therapeutic development.

miRNA	Disease	Stage of clinical trial	Reference
miRNA replacement			
*let-7*	Lung cancer	Preclinical	[106]
miR-34	Lung cancer, Prostate cancer	Preclinical	[107,108]
miR-29	Cardiac fibrosis	Preclinical	[109]
miRNA antagonists			
miR-122	Hepatitis C virus	II	[110]
miR-208/499	Chronic heart failure	Preclinical	[111]
miR-15/195	Post-myocardial infarction remodeling	Preclinical	[112,113]
miR-206	Amyotrophic lateral sclerosis	Preclinical	[114]
miR-451	Myeloproliferative diseases	Preclinical	[115]

#### 3.2.1. Role of microRNA in Inflammatory Lung Diseases

There is evidence that upregulation or downregulation of miRNAs is critical for lung homeostasis and, thus, may contribute to the development of pathological pulmonary conditions. Many studies have focused on the role of miRNAs in inflammatory lung diseases, such as COPD [116,117], pulmonary fibrosis [118,119,120,121], and asthma [122,123,124,125] (Table 3).

**Table 3:** miRNAs in inflammatory lung diseases.

Lung diseases	Expression of specific miRNA		Reference
COPD	miR-223/1274a	↑	[126]
	*let-7*	↓	[117]
	miR-1	↓	[116]
	miR-146a	↓	[127]
	miR-150	↓	[117]
Asthma	miR-21	↑	[123]
	miR-126	↑	[124]
	miR-155	↑	[122]
	miR-133a	↓	[125]
Pulmonary fibrosis	miR-21	↑	[118]
	miR-155	↑	[128]
	*let-7d*	↓	[119]
	miR-29	↓	[120]
	miR-200	↓	[121]
Smoking-related miRNA	*let-7*	↓	[129]
	miR-10a	↓	[129]
	miR-34	↓	[129]
	miR-123	↓	[129]
	miR-145	↓	[129]
	miR-150	↓	[117]
	miR-199b	↓	[130]
	miR-218	↓	[130]
	miR-222	↓	[117,129]

The pathogenesis of COPD is attributed to not only chronic inflammation in the airways but also systemic inflammation [131]. Cigarette smoking is the main risk factor for the development of COPD. Smoking has been shown to cause biological change in the gene expression of the lungs [132], and there are some reports about smoking-related miRNAs [117,129,130]. However, there are few reports that focus on the miRNAs related to the pathogenesis of this disease with systemic inflammatory components. Recent study on pulmonary fibroblasts of COPD patients presents less expression of miR-146a after stimulation with proinflammatory cytokines when compared with non-COPD subjects with similar smoking histories [127]. The downregulation of miR-146a resulted in a prolonged mRNA half-life of cyclooxygenase-2, thus increasing prostaglandin E2 in fibroblasts from COPD subjects. Moreover, Ezzie *et al.* researched the difference of miRNA profiles expressed in the lungs of smokers with and without COPD. They concluded that miR-223 and miR-1274a were the most affected miRNAs in subjects with COPD [126]. Yet, COPD is a complex, multi-component, and heterogeneous disorder with a number of different pathological processes and subgroups with their own characteristics and natural history [133]. A better understanding of the complexity of the disease and potential clinical relevance of the identified miRNAs is needed.

Pulmonary fibrosis can be caused by an identifiable irritation to the lungs, but, in many cases, the cause is unknown, and the therapeutic possibilities are limited. Cigarette smoking is one of the most recognized risk factors for the development of pulmonary fibrosis. This disorder is mainly accompanied by increased expression of the key fibrotic mediator transforming growth factor β (TGF-β) and other cytokines produced at the lesion of active fibrosis [128]. Recently, it was reported that miRNAs may play an important regulatory role in the pulmonary fibrotic change in the lungs. The downregulation of *let-7d* in idiopathic pulmonary fibrosis (IPF) resulted in increased collagen deposition and alveolar septal thickening [119]. In addition, Liu *et al.* reported that the oncogenic miR-21 was found to be upregulated in IPF patients and in the murine lungs with bleomycin-induced fibrosis [118]. Although these miRNAs may be potential therapeutic targets because their expression is related to the regulation of TGF-β, the factor is necessary but not sufficient for pathologic fibrosis of the lungs. Pulmonary fibrosis is also a complicated illness that can have many different causes.

Focus on the role of miRNAs in asthma has recently increased. Asthma is an inflammatory disease of the airway that is characterized by an abnormal response of T helper-2 (Th2)-type CD4+T lymphocytes against inhaled allergens [134]. In a different asthmatic mouse model, there was an observed increase in the expression of miR-21 in the lungs [123]. This report might contribute to the understanding of the inflammatory mechanism in the airway through the inhibition of IL-12, favoring the Th2 lymphocyte response. A toll-like receptor 4 (TLR4)-induced Th2 lymphocyte induces high expression of miR-126, and selective blockade of miR-126 suppressed the asthmatic phenotype [124]. In addition, airway remodeling is a characteristic feature of asthma and has important functional implications. Rodriguez *et al.* have shown that miR-155 is related to the development of inflammatory infiltration into the lung and airway remodeling [122]. Thus, some studies present a functional connection between miRNA expression and asthma pathogenesis and suggest that targeting miRNAs in the airways may lead to anti-inflammatory treatments for allergic asthma. Despite the evidence from experimental models, the expression profiling of miRNAs in airway biopsies from patients with mild asthma before and after treatment with inhaled corticosteroids and in healthy volunteers revealed no differences in miRNA expression [135]. Further investigations about the role of miRNAs related to asthma pathogenesis are required.

Although the basic evidence of miRNA biology is still providing new insights, applications of miRNA-based therapy for inflammatory lung diseases are less advanced than those for lung cancer [136]. One reason for this could be that the disease heterogeneity is caused by the effects of many environmental air pollutants, including smoke and volatile organic compounds. The presence of several risk factors makes the understanding of the pathogenesis of inflammatory lung diseases complicated. Understanding the role that miRNAs play in the modulation of gene expression, leading to sustain the pathogenesis of lung diseases, is important for the development of new therapies that focus on the prevention of disease progression and symptom relief.

#### 3.2.2. microRNA/Anti-microRNA Delivery for Lung Cancer Therapy

Given the significant roles that miRNAs play in multiple pathways of lung carcinogenesis, increasing efforts are dedicated to the research and development of miRNA-based therapies, including restoring functions of tumor suppressive miRNAs or inhibiting oncogenic miRNAs. The development of miRNA-based therapies for lung cancer is growing prosperously with the help of new RNAi technologies. Compared to siRNA-based therapies, which are already in clinical trials, miRNAs are less toxic and have the potential to target multiple genes. The difficulty associated with miRNA delivery is mainly equal to that of siRNAs. The critical problems for the development of this therapy are effective delivery into target sites, potency of the therapy, and elimination of off-target effects [137].

There are two strategies as the therapeutic applications of miRNAs for lung cancer [138]. One strategy is miRNA replacement therapy, which involves the re-introduction of a tumor suppressor miRNA mimic to restore a loss of the function. MiRNA mimics are synthetic RNA duplexes designed to mimic the endogenous functions of miRNAs with chemical modifications for stability and cellular uptake. The concept of miRNA replacement therapy is most exemplified by the *let-7* miRNA. *let-7* is a tumor-suppressor miRNA in non-small-cell lung cancer that inversely correlates with the expression of the RAS oncoprotein, a key cancer gene [139]. Intranasal administration of *let-7* mimic into mouse models of lung cancer significantly reduced tumor growth, suggesting that miRNA replacement therapy is indeed promising [106,140,141]. Another miRNA that shows the value of miRNA replacement is provided by miR-34a [107,142]. Local and/or systemic delivery of a synthetic miR-34a mimic led to accumulation of miR-34a in the tumor tissue and inhibition of lung tumor growth. Lately, Ling *et al.* also showed that tumor suppressor miR-22 exhibited anti-lung cancer activity through post-transcriptional regulation of ErbB3 [143]. Thus, therapeutic miRNA mimics have a powerful potential by attacking multiple genes relevant to several diseases. However, it is necessary to pay attention to the potential toxicity in normal tissues under conditions in which the therapeutic delivery of miRNA mimics will lead to an accumulation of exogenous miRNAs in normal cells [138]. Although the assumptions are well founded, there is still insufficient evidence for toxicity caused by miRNA mimics. Indeed, several *in vivo* studies failed to reveal side effects caused by the miRNA mimics and suggested that delivery of miRNA mimics to normal tissues was well tolerated [107,141]. It will be important to research miRNA mimic-induced effects in normal cells and to carefully assess toxicity before using them in clinical practice.

The second strategy is directed toward a gain of function and aims to inhibit oncomiRs by using anti-miRNAs. Chemical modifications, such as 2'-*O*-methyl-group and locked nucleic acid (LNA), would increase oligo stability against nucleases [144]. Antisense oligonucleotides contained in these modifications are termed antagomirs or “LNA-antimiRs” [144,145]. They are oligonucleotides with sequences complementary to the endogenous miRNA and inhibit the specific miRNA function. An LNA-antimiR against miR-122 has been shown to effectively silence miR-122 in non-human primates [145], and the findings support the potential of these compounds as a new class of therapeutics. Moreover, it has also been reported that anti-miR-150 delivered into lung tumor xenografts in mice led to inhibited tumor growth [146]. Relative to studies on miRNA mimics, studies with antisense oligonucleotides have shown effective evidence with naked oligonucleotides. This illustrates the potential of chemical modifications of oligonucleotides to improve their stability, resistance to RNase, and pharmacologic properties. Therefore, inhibition of miRNA function by chemically modified antimiR oligonucleotides has become an important and widely used approach. Recent data from the first phase II study in patients with chronic HCV infection treated with the LNA-modified antimiR-122 showed that this compound was well tolerated and provided continuing viral suppression.

An increasing number of studies have examined the therapeutic potential of miRNAs. Recently, the evidence of roles for miRNAs in determining drug resistance has emerged [147]. Cytotoxic and molecular target drugs have been widely used in the treatment of advanced lung cancer; unfortunately, many cases are still refractory to chemotherapy. In this situation, combining miRNA mimics or antimiR with chemotherapy may potentiate the efficacy of the cancer treatment in the future. In addition, miRNAs related with cancer stem cells may significantly broaden the field of miRNA-based therapy and suggest that miRNAs can be potential tools to kill cancer cells associated with therapy resistance, recurrence, and metastasis [108,148]. Hence, the main challenge is the successful delivery and chemical modifications of the therapeutic miRNAs to the target tissue without harming normal tissues.

## 4. Conclusions and Prospects

RNAi-based approaches provide a promising therapeutic modality for the treatment of various lung diseases. One of the greatest challenges in RNAi-based therapy continues to be the delivery method of the therapeutic siRNAs and miRNAs to the target cells. Pulmonary delivery applications are very attractive, since they tend to be non-invasive, are locally restricted, and can be administered by the patient. A realistic therapeutic intervention, such as aerosolization, can enhance drug delivery to the site of action and decrease systemic exposure of the patient to the therapy, thereby reducing off-target effects. The advancement of pulmonary siRNA delivery to the clinic illustrates that RNAi-based therapy holds a central place in the future treatment of lung diseases. On the other hand, miRNAs have the opportunity to target multiple genes in a fine-tuned manner, and the miRNA-based therapy will provide an attractive anti-tumor and anti-inflammatory approach for various lung diseases. In particular, anti-miRNA therapy by chemically modified antimiR oligonucleotides has become a potential therapy for lung diseases because the oligonucleotides can be successfully delivered without delivery vectors. Increased evidence has indicated that miRNAs fulfill causative roles in a variety of lung diseases and have prompted investigations into their potential as therapeutic targets. Further understanding of the detailed mechanisms of RNAi-based therapy and investigations of more effective delivery methods are required for future development. These novel approaches could open new avenues for various lung diseases and improve the clinical outcome of the patients.
